# Analysis of the Memoirs of the Medical Society of London

**Published:** 1799-08

**Authors:** 


### *Analyſis of the Memoirs of the Medical Society of London, Vol. V. 8vo. pp.* 470. Johnſon, *London*, 1799.

THE preceding volumes of the Memoirs of this Society, which have been regularly continued ſince the year 1786, are too well known to medical readers to require any account of them in this place. The preſent volume appears appears in no respect inferior to the preceding ones: it contains about forty articles of original communications, among which the following appeared to us particularly connected with practical utility:—

I. A case of hydrophobia, by William Gaitskell, surgeon.

The internal and external use of oil was tried in this case, without success: the patient, however, is said to have lived to the eighth day from the appearance of hydrophobia, which is nearly double the usual time.

V. This article contains an account of the efficacy of the Zanthoxylon, both externally and internally applied.

The following account of the tree and its uses is supplied by Mr. W. Chamberlaine, surgeon, F.M.S. and secretary to the Medical Society:—

"A botanical account of the Zanthoxylon, with an accurate drawing of it, and the history of several cases in which it had succeeded beyond expectation, Dr. Harris assures me, he committed to the care of a confidential friend, to be delivered to me for the purpose of being laid before the Medical Society; but the ship by which these articles were sent was captured.

"The part used is a powder from the bark of the root of a tree, which, from specimens of the root now in my possession, and from every other document, appears to be that called Prickly Yellow Wood, or Yellow Hercules, much used for making the heading of sugar hogsheads, for bedsteads, and many other purposes in Jamaica; it is a tall, straight tree, easily distinguishable from all others, in appearance not greatly dissimilar to our ash; the bark of the trunk is thickly beset with prickles, which, in the young trees, are pointed, but obtuse in the more aged. The wood is of a very bright yellow colour, whence its name *Zanthoxylon*. The flowers, which bear some resemblance to the cassia fistula, are succeeded by a pod, flattish, and not much unlike in shape and size to a man's thumb; this pod is at first green, then red, and lastly turns black when ripe, and contains three or four compressed seeds.

"It is called by Browne*, *Zanthoxylum foliis oblongis obovatis* pinnatis *et leviter crenatis*; *floribus racemosis*, *caudice spinoso*, *ligno subcroceo*.

"Dr. Harris, by a former ship, sent me a small box of the powder, which I have had opportunities of trying in three or four cases of very bad ulcers of the legs. In the first I had attended the patient from January to July, with very little success; but, on changing the dressings for the powder of Zanthoxylon, an almost immediate alteration took place; the wound was quite healed up by the end of September, and the patient has continued ever since perfectly well.

"My

* Nat. Hist. Jamaica, Sect. 4. *Pentandria Pentagynia*.

" My second patient had been in an hospital six weeks in May and June, and was discharged cured, but the ulcer soon after broke out again, and continued to increase to the extent of five inches and a half in length, and four in breadth, which were its dimensions when I was first called to look at it; I ordered that it should be fomented, and after washing it with milk and water, sprinkled the powder liberally all over the surface of the ulcer, and covered the whole with plantain leaves, rejecting all greasy ointments. This was persisted in twice a day, and a cataplasm of bread and milk laid over all, at each dressing. Internally she took hydrarg. cum sulph. et nitr. āā gr. xv. bis die. In a week the ulcer was less than half its former size, looked perfectly clean, and put on every appearance that could be wished for, and soon became perfectly well.

" A poor woman had one of the worst ulcers I ever saw; she was very much reduced through want of sleep, from the excruciating pain caused by the ulcer, which she suffered night and day, without intermission. I gave her some of the Zanthoxylon powder, and instructed her how to use it. In five days she walked to my house, and was able to come every morning to be dressed. I gave her no medicines, and enjoined no regimen, but the leg was perfectly healed by the application of the Zanthoxylon alone, in a month, and she now follows her business of a laundress, no vestige of any ulcer remaining, except a little redness."

### *On the Efficacy of the Zanthoxylon.*

*By* Thomas Heney, *M.D. of St. David's, in the Island of Jamaica.*

[Communicated by John Harris, M.D. of Kingston, Jamaica, C.M.S.]

Read January 20th, 1794.

*To Dr. Harris.*

" Dear Sir, *St. David's, Sept.* 12, 1792.

" I regret that the limits of a letter will not give you a minute account of the success of my experiments with the Zanthoxylon. The first intimation of its virtues I owe to you; and at the same time witnessed its surprising efficacy in the case of Mr. G——n's hæmatocele. However strong my reliance on your account of it at that period, and on the conviction of my own senses in Mr. G——n's case, yet the deceptions practised in the world by the exaggerated accounts of the *cicuta*, the *flammula jovis*, the *arnica*, the *digitalis purpurea*, and others, by authors of no inconsiderable name in the medical world, had infused so large a portion of the sceptic through my medical creed, that I determined nothing less than repeated *autopsia* should convince me. I therefore instituted my experiments with as unprejudiced and candid a mind as ever a son of Hippocrates did. I must sincerely own, I lost sight of the warning of that great master, " Experimentum periculosum; " but never of the preceding text, " Judicium difficile," until, by repeated experience, I had the fullest conviction of its efficacy in numberless cases, and principally those I shall mention.

" You

"You well know how difficult it is to bring the foul ulcers, to which the unhappy children of Africa are in this climate subject, to such a condition as promises a speedy cure, particularly such as are attended with loss of substance, and how frequently we are obliged to have recourse to the unpleasant use of escharotics, to remove those fungous excrescences which the habitual nastiness of the Negroes, and the irritation of insects (which in tropical countries inhabit every broken particle of animal and vegetable substances), produce. To remedy the inconveniences arising from these causes, I determined to make trial of the Zanthoxylon, in manner and form as we had done in Mr. G——n's case. I therefore laid aside the tinctures of myrrh and guaiacum, Hungary water, phagædenic water, lime water, and every other usual application, and commenced the use of the Zanthoxylon, by bathing the ulcers with the decoction of that bark, and intermixing it with the dressings.

"For the purpose of more minutely ascertaining its efficacy, I confined the patients thus treated in the same place, with others whose ulcers were of the same date and condition, and whom I treated with the lotions, dressings, and poultices in common use.

"In addition to the external use of the Zanthoxylon, I ordered a couple of ounces of its bark to be boiled with the sarsaparilla, in lieu of the lignum vitæ, for their drink.

"The ulcers of my Zanthoxylon patients, in the course of a few days, invariably threw off the sloughs, and other foul appearances, and exhibited healthy and well-coloured granulations beneath, discharging laudable and well-conditioned pus. Their co-patients, whether treated with mild emollient applications, or with stimulant dressings, exhibited in their several situations such slow appearances of amendment, as finally urged me to use the Zanthoxylon to all.

"A Negro woman, who had been affected for many years with several large phagædenic ulcers, from the mid-thigh to the ancle, was put under my care. A fetid, sanious discharge, together with fungous, and almost gangrenous excrescences, had given to the ulcerated surface so horrid an appearance and stench, as was highly disgusting to every one who saw or approached it, and intolerable to the wretched patient herself.

"For six weeks after the first inspection of the ulcers, escharotics, warm stimulating dressings, tight bandages, were tried to no purpose. In place of these I commenced the use of the Zanthoxylon, by bathing the sores with the decoction, intermixing the powdered bark in the dressings, and giving the bark in decoction, in the place and proportion of the lignum guaiacum; the events answered my expectations, the discharge soon acquired the condition of laudable pus; well-coloured granulations, in the happiest form, appeared, and saturnine ointments finally effected the cure in eight weeks. Numberless were the experiments made by me and my assistant subsequent to this, in similar ulcers, with equal success.

In every inftance, however, of venereal taint in yaws, or crab yaws, I found it ineffectual, prior to the ufe of mercurials.

"I fome time ago mentioned to you a few fuccefsful experiments, made with a view to determine its anti-febrile qualities, by adminiftering it in the fame fcope and indication as the Peruvian bark. Repeated trials have, however, fince that period, convinced me, it is much more inactive than that celebrated febrifuge, unlefs its virtues are fharpened by the addition of fome neutral falt or alcaline; then it really exhibits virtues little inferior to the China-China, and is unattended with the inconveniences ufually experienced from the latter. This I account for by fuppofing its refinous parts to be rendered more readily mifcible with the aqueous juices, by the addition of the falt.

"Another moft fingular quality the Zanthoxylon poffeffes in an eminent degree, which I prefume you are unacquainted with, and to the knowledge of which I was accidentally led. A fhort account of the difcovery will beft explain it, and, at the fame time, indirectly argues this falutary plant not to be a native of Jamaica.

"Mr Crofdale purchafed two negro wenches in the beginning of the prefent year; the younger of whom, at different times fince, has been afflicted with a dry belly-ach, or colica pictonum. About two months ago fhe was feized with it in fo dreadful a degree, that every effort to remove the fpafmodic conftriction of the bowels, and procure fome motions, proved ineffectual. To no purpofe were emollient fomentations, anodyne, or cathartic glyfters, mild and draftic purges, caftor oil, and ultimately, blifters to the abdomen, applied. That horrid fymptom, a *vomiting of the excrements*, commenced, and banifhed every ray of hope. In this fituation fhe defired to have her fifter with her, who, on feeing her deplorable condition, fignified a wifh of giving a noftrum communicated to her by their mother, and employed to cure herfelf, on a fimilar ocafion, in Africa. I readily complied with the requeft. In the courfe of two hours, fhe returned from the woods with the root and flowers of fome plant, pounded together in a calabafh. Two fpoonfuls of the expreffed juice of this fhe gave her fifter twice, at an interval of two hours each. The firft effect of this was a tranquil, profound fleep, of twelve hours duration, during which the pulfe and breathing gradually returned to the natural ftate; after this, all fenfe of pain, and every bad fymptom, difappeared, and no other inconvenience did fhe experience, fave debility, and flight forenefs, from the paffing of the purgative medicine, which came away efpecially during the courfe of the following day. The fifter was obferved to boil the ingredients (after expreffing the juice) in a large quantity of water, and give it on the following day as common drink. No reward or menace could induce her to difcover the plants, until ftratagem brought it to light. We induced another negro to diffemble a fimilar complaint, and prevailed with the wench to feek for, and prepare the fame cure; in complying with this requeſt, we had her ſo narrowly watched, as to diſcover the ſecret to be the freſh root of the zanthoxylon, in its infant ſtate, intermixed with the ſaffron-coloured flower of the wild ſage; which laſt I have ſince found to contribute nothing to its virtues. Having procured ſome of the ſappy and ſmalleſt roots of the young trees, and expreſſed the juice, I began the experiment of its qualities on myſelf, at tea-ſpoonful doſes. From the firſt of theſe, I found no other effects than an unuſual flow of ſpirits. By continuing the doſe, drowſineſs, nauſea, head-ach, and, at length, ſleep enſued; from which I however awoke next morning perfectly refreſhed, and had three copious eaſy motions. I preſerved ſome of the juice with rum, and ſome with ſyrup. Theſe preparations, as well as the juice, I have frequently ſince that period adminiſtered in complaints of the bowels (ſo frequent among the African race and their progeny) with every wiſhed-for ſucceſs. On the eſtate of Mrs. O'Bryan, an old man of eighty years, was lately ſeized with convulſive fits every hour, in every character ſimilar to epilepſy, which continued, without intermiſſion, twenty-four hours. To him, on being ſent for, I immediately gave a wine-glaſsful of the juice preſerved in rum; the fit which ſucceeded the firſt glaſs was unattended with ſtrong convulſions, and the ſecond was little elſe than a comatoſe ſtate; after which, a ſound ſleep of ten hours removed every appearance of diſorder, except laſſitude.

"This laſt mentioned anti-ſpaſmodic virtue the Zanthoxylon loſes by being dried and powdered, its narcotic qualities being diſſipated with the moiſture of the plant.

"Theſe are the chief remarks my opportunities and leiſure have hitherto enabled me to make on the Zanthoxylon. I ſhall continue to give it every candid and fair trial, and, from time to time, will ſend you the reſults of my experiments. Some other vegetables have fallen under my inſpection from negro information, and I have really found much ſatisfaction from their uſe. My botanic information is too limited to attempt their deſcription; yet, as ſoon as I learn it, you ſhall alſo know them and their virtues.

"I remain, dear Sir,

"Your obliged humble Servant,

"THOMAS HENEY."

VIII. Several caſes of the lithontriptic power of the muriatic acid, when exhibited in doſes of ten to twenty drops or more in a glaſs of water, three times a day, are given by Mr. Copland.

IX. Experiments on the external uſe of the tartarized antimony, by B. Hutchinson, ſurgeon.

In theſe experiments Mr. Hutchinſon obſerved, that when the palms of the hands were waſhed with the ſolution of emetic tartar, it produced a ſoporific rific effect, and increased the secretion of the skin and bladder. The quantity washed in was from five to fifteen grains, at bed-time,

By washing in fifteen grains night and morning, for ten days, a most obstinate tertian was cured, but the patient complained of *constant sickness*, after the second day of using it.

No mention is made of any eruption being produced by it in this instance; in several others the usual eruption is mentioned, and the production of nausea.

XIII. Contains an excellent account of the Harrowgate waters, by Dr. Garnett, for the analysis of which we are obliged to refer to the volume itself.

(To be continued in our next Number.)

